# Exclusive human milk diet and mortality before discharge in premature and very low birthweight infants: a systematic review and meta-analysis

**DOI:** 10.3389/fnut.2026.1898548

**Published:** 2026-08-21

**Authors:** Sarah M. Reyes, Tristen L. Paul, Jenelle Ferry

**Affiliations:** 1 Rev Bioscience, LLC, Boise, ID, United States; 2 Department of Neonatology, Pediatrix Medical Group Tampa Regional Practices, St. Joseph’s Women’s Hospital, Tampa, FL, United States

**Keywords:** exclusive human milk diet, human milk fortifier, meta-analysis, mortality, very low birthweight infants

## Abstract

**Background:**

Survival to hospital discharge remains a critical outcome for premature and very low birthweight infants (VLBW), who often require fortification of human milk to meet nutritional needs within tolerated feeding volumes. Although exclusive human milk diets (EHMD) that include human milk-derived fortifiers have been proposed as an alternative to cow milk-derived fortification or formula supplementation, evidence for their association with improved survival remains inconsistent across studies.

**Objective:**

To evaluate the association between an EHMD and survival to hospital discharge among premature and low birthweight infants, synthesizing evidence from randomized and non-randomized studies.

**Methods:**

PubMed, Scopus, and Web of Science were searched through July 2025 for studies comparing an EHMD (human milk fortified with human milk-derived fortifier) with diets containing cow milk-derived products. Two reviewers independently screened studies, extracted data, and assessed risk of bias. Pooled odds ratios (OR) were calculated using random-effects meta-analysis, with subgroup analyses by study design.

**Results:**

Five randomized and 9 non-randomized studies were included, encompassing 4,713 infants born between 2004 and 2021. Mean birthweights ranged from 741 to 1,361 g, and mean gestational ages ranged from 25.5 to 29.7 weeks. In pooled analyses, EHMD was associated with lower odds of death before discharge (pooled OR 0.80, 95% CI 0.65–0.98; *I*^2^ = 0%, tau^2^ = 0.00, *p* = 0.03). Directionally similar associations were observed in randomized (OR 0.62, 95% CI 0.28–1.38) and non-randomized studies (OR 0.82, 95% CI 0.66–1.03), although no significant differences emerged within either subgroup. In the two randomized trials directly comparing human milk-derived and cow milk-derived fortifiers, mortality estimates were directionally similar but no significant difference was observed between groups.

**Conclusion:**

EHMD was associated with lower odds of death before discharge in pooled analyses. Although no significant differences emerged in the subgroup analyses, the consistency in effect direction across study designs supports the possibility of a clinically meaningful association. Adequately powered randomized and pragmatic trials are needed to confirm this association.

**Systematic review registration:**

https://www.crd.york.ac.uk/PROSPERO/view/CRD420251059764, PROSPERO: CRD420251059764

## 1 Introduction

Survival to hospital discharge remains a key outcome among premature and very low birthweight (VLBW) infants, whose risk of mortality increases progressively with decreasing gestational age and birthweight (1, 2). Human milk feeding is associated with improved survival in this population, likely through its bioactive components, which modulate inflammatory responses, support gastrointestinal maturation, and promote a favorable gut microbiome (3). However, premature infants, particularly those born extremely preterm or with very low birthweight (<1,500 g), have greater nutritional requirements than term infants (4). Although human milk is the preferred choice for enteral feedings, its nutrient content alone is often insufficient to meet these requirements within tolerated feeding volumes, leading to recommendations for human milk fortification (3, 5).

Recognition of the clinical benefits of human milk prompted interest in human milk-derived fortifiers as an alternative to conventional cow milk-derived fortifiers and formula supplementation. Some randomized and observational studies have reported improved survival among infants receiving an exclusive human milk diet (EHMD, human milk fortified with human milk-derived fortifiers) compared with diets containing cow milk-derived fortifiers or formula (6–8). However, others have not demonstrated clear associations (9, 10). Differences in findings may reflect variation in control group diets, timing and advancement of fortification, degree of human milk exposure, and other clinical or methodological factors. Such variability is common in neonatal nutrition research, where feeding protocols differ across institutions and evolve over time alongside broader advances in neonatal care and quality improvement initiatives that may independently influence mortality outcomes. A clearer understanding of potential differences between human milk-derived and cow milk-derived fortification strategies is needed to inform clinical decision-making.

Evaluating the effects of nutritional interventions on mortality in preterm and VLBW infants presents important methodological challenges because death before discharge, while clinically serious, remains a relatively uncommon outcome (11). At the same time, most clinical trials in this population enroll relatively small numbers of participants. Consequently, individual trials may lack sufficient statistical power to detect clinically meaningful differences in mortality. When outcomes are uncommon and large trials are difficult and expensive to conduct, reliance on a single study design may provide an incomplete assessment of associations. Contemporary evidence frameworks increasingly recognize that randomized trials and well-conducted non-randomized studies provide complementary insights (12). Integrating evidence across study designs may therefore help characterize both efficacy under controlled conditions and effectiveness in routine clinical practice (13). Although observational studies remain susceptible to bias, including residual confounding related to clinical indication and center-level feeding practices, structured risk-of-bias assessment and transparent synthesis can help contextualize their contribution to understanding real-world clinical outcomes (12, 14).

To address these considerations, we synthesized the available evidence comparing EHMD with cow milk-containing diets in premature and VLBW infants. Our objective was to evaluate the association between EHMD and survival to hospital discharge while exploring potential sources of heterogeneity across studies.

## 2 Methods

This systematic review and meta-analysis was conducted in accordance with the Preferred Reporting Items for Systematic Review (PRISMA) guidelines (15) and was registered with PROSPERO (CRD420251059764).

### 2.1 Search strategy and screening

PubMed, Scopus, and Web of Science were searched for relevant English-language articles through July 2025. Search terms combined controlled vocabulary and keywords related to human milk, human milk fortifiers, prematurity, low birthweight, NEC and mortality. The full search strategy is provided Supplementary Table 1. Search results were imported into Covidence (Veritas Health Innovation, Melbourne, Australia), and duplicates were removed automatically by the software or manually. Records were then screened in duplicate using Covidence, which maintains reviewer decisions separately to minimize bias. Conflicts were resolved by consensus.

### 2.2 Study selection criteria

We included randomized controlled trials (RCTs) and non-randomized cohort studies investigating an EHMD compared with cow milk-based nutrition on survival to hospital discharge among infants born ≤37 weeks’ gestation or <1,500 g at birth. An EHMD was defined as vat-pasteurized, human milk-derived human milk fortifiers added to a base diet of mother’s own milk (MOM) and/or donor human milk (DHM). The comparison group consisted of infants who received cow milk-derived nutritional products, including cow milk-derived human milk fortifiers and/or infant formula, as reported by study authors.

Studies were excluded if they were: (1) studies of special populations (e.g., surgical infants); (2) studies lacking a comparator group receiving cow milk-derived products; (3) non-human studies; (4) case studies, (5) reviews, commentaries, letters to the editor, and conference proceedings; (6) no relevant outcomes reported or obtained after author contact (Supplementary Table 2). To prevent double-counting patients, we assessed potential cohort overlap by reviewing study setting, enrollment periods, participating centers, sample sizes, and author groups. When overlap was unclear, study authors were contacted for clarification. In cases of confirmed or likely overlap, only the most comprehensive or relevant dataset was included, and linked or secondary analyses were excluded.

### 2.3 Data extraction

Two reviewers independently collected data using a standardized form. Variables were collected at either the study level (e.g., bibliographic details, study design) and the group level (e.g., fortification methods, outcomes). Conflicts were resolved by consensus.

### 2.4 Outcome measures and data harmonization

Our primary outcome was mortality before discharge, as defined by study authors. Mortality reporting across studies varied, with some authors reporting terms such as mortality, death, and survival until discharge. Mortality data were extracted as reported event counts within each study group.

Exposure variables reflected enteral nutrition during the period when an EHMD is typically administered, generally from birth to approximately 34 weeks’ postmenstrual age or an enteral intake of 150–180 mL/kg/d (Table 1). The EHMD group included infants who received human milk fortified with vat-pasteurized human milk-derived fortifier and no exposure to cow milk-derived nutritional products during this defined feeding window, regardless of specific fortification protocols and as reported by study authors. The control group included infants who received any cow milk-derived nutritional products during the same period, including cow milk-based fortifier and/or infant formula. This definition reflects variability in contemporary NICU feeding practices and was intended to evaluate outcomes associated with elimination versus exposure to cow milk-derived products during the early feeding period. After this period, fortification practices may vary and were not considered part of the exposure definition.

**Table 1:** Summary of included studies.

Publication and location	Participant birth year	Characteristics of included participants		Diet and fortification		Sample size EHMD/Control
		EHMD	Control	EHMD* fortification	Control diet	
Non-randomized studies						
Bushati et al. (22)	2018–2019	BW: 821.7 ± 110 GA: 28 ± 3	BW: 741.0 ± 150 GA: 26 ± 2	6 kcal/oz. @ 60 mL/kg/d; 8 kcal/oz. at 120 mL/kg/d; hmCR if weight gain < 15 g/kg/d on 8 kcal/oz	MOM + CMBF @ 2 kcal/oz. at 80 mL/kg/d; 4 kcal/oz. at 100 mL/kg/d**	15/49
Carome et al. (23)	2012–2017	BW: 770 (630–880) GA: 25.5 ± 1.8	BW: 770 (660–880) GA: 25.7 ± 1.9	4 kcal/oz. @ 80 mL/kg/d; then 6–8 kcal/oz. if growth <15 g/kg/d	MOM + CMBF @ 2 kcal/oz. at 80 mL/kg/d; 4 kcal/oz. 1 day after initiation then 6 kcal/oz. if growth <15 g/kg/d	127/179
Eibensteiner et al. (24)	2012–2018	BW: 752 (659, 893) GA: 26 + 1 (24 + 6, 27 + 1)	BW: 773 (650, 890) GA: 25 + 6 (24 + 5, 27 + 3)	6 kcal/oz. @ 100 mL/kg/d	MOM + CMBF @ 4.4 kcal/oz. at 100 mL/kg/d or preterm infant formula	96/96
Hair et al. (7)	2006–2014	BW: 844 ± 210 GA: 26.5 ± 2.5	BW: 823 ± 205 GA: 26.4 ± 2.3	60 mL/kg/d or 100–120 mL/kg/d	MOM+ CMBF and/or preterm infant formula	819/768
Hanford et al. (25)	2016–2018	BW: 1,019.3 ± 285.5 GA: 27.6 ± 1.83	BW: 1,055.4 ± 258.2 GA: 27.3 ± 1.59	4–8 kcal/oz. @ 80 mL/kg/d depending on growth	MOM + CMBF @ 80 mL/kg/d or preterm infant formula depending on growth	53/36
Harris et al. (21)	Not reported	BW: 831.9 ± 196.4 GA: 27.2 ± 2.44	BW: 834.6 ± 198.9 GA: 27.2 ± 7.42	4 kcal/oz. @ 40 mL/kg/d	MOM/DHM + CMBF	105/95
Herrmann and Carroll (26)	2004–2012	BW: 1,361 ± 542 GA: 29.6 ± 3.0	BW: 1,334 ± 0.436 GA: 29.7 ± 2.5	Not reported	MOM + CMBF start/advancement not reported	199/443
Sato et al. (9)	2012–2018	BW: 1,239 ± 154 GA: 29.5 ± 2.5	BW: 1,269 ± 144 GA: 29.6 ± 2.1	4 kcal/oz. @ 90 mL/kg/d	MOM+ CMBF @ 4 kcal/oz. at 90 mL/kg/d	265/134
Wickland et al. (27)	2013–2016	BW: 890.0 (858.8–931.2) GA: 27.1 (26.7–27.8)	BW: 940.0 (887.9–972.1) GA: 27.6 (27.1–27.9)	4 kcal/oz. @ 80 mL/kg/day; increased to 6 kcal/oz. if growth <20 g/kg/d	MOM/DHM + CMBF 4 kcal/oz. @ 80 mL/kg/day	275/218
Randomized studies						
Cristofalo et al. (28)	2007–2008	BW: 996 ± 152 GA: 27.7 ± 1.5	BW: 983 ± 207 GA: 27.5 ± 2.4	40 mL/kg/d or 100 mL/kg/d	Preterm infant formula	29/24
Embleton et al. (29)	2017–2019	BW: 930 (733, 1,095) GA: 27.1 (25.7, 28.1)	BW: 910 (704, 1,054) GA: 27.0 (26.0, 28.1)	6 kcal @ 150 mL/kg/d	MOM + preterm formula @ 150 mL/kg/d	63/63
Jensen et al. (30)	2019–2021	BW: 793 (212) GA: 25.6 (24.6, 26.7)	BW: 787 (207) GA: 26.0 (24.5, 27.1)	6 kcal @ 150 mL/kg/d	CMBF	115/113
O’Connor et al. (10)	2014–2016	BW: 887 ± 208 GA: 27.9 ± 2.7	BW: 889 ± 196 GA: 27.5 ± 2.3	4 kcal/oz. @ 100 mL/kg/d; 6 kcal @ 140 mL/kg/d, then 8 kcal if weight gain <15 g/kg/d after achieving full feeds (160 mL/kg/d)	MOM + CMBF @ 2 kcal/oz. at 100 mL/kg/d; 4 kcal/oz. at 140 mL/kg/d, then 6 kcal/oz. if weight gain <15 g/kg/d aft er achieving full feeds	64/63
Sullivan et al. (31)	2007–2008	BW: HM100: 945 ± 202 GA: HM100: 27.2 ± 2.2 BW: HM40: 909 ± 193 GA: HM40: 27.1 ± 2.3	BW: 922 ± 197 GA: 27.3 ± 2.0	40 mL/kg/d or 100 mL/kg/d	MOM + CMBD @ 100 mL/kg/d or preterm infant formula	138/69

Values represent mean ± SD or median (IQR), as reported by study authors. *EHMD is created by adding human milk-derived fortifier to donor or maternal milk. In all cases, the human milk-derived fortifier used was vat-pasteurized and manufactured by Prolacta Bioscience.**Liquid protein fortifier and microlipids if inadequate growth. CMBF, cow milk-based fortifier; DHM, donor human milk; hmCR, human milk; MOM mother’s own milk.

Other variables collected included participant characteristics (e.g., birthweight and gestational age at birth) and bibliographic details (e.g., study enrollment years, geographic region, and study design).

### 2.5 Quality assessment

Two reviewers independently performed risk of bias assessments for all included studies. Disagreements resolved through discussion or adjudication by a third reviewer. Non-randomized studies were evaluated using the RoBNObs tool (16), developed and used in the 2020 Dietary Guidelines for Americans (17). Domains included confounding, participant selection, exposure classification, deviations from intended exposure, missing data, outcome measurement, and selective reporting. Each domain was rated as low, moderate, serious, critical, or no information. Overall risk was assigned based on the highest domain score or through cumulative downgrading when multiple moderate or serious ratings were present.

Randomized trials were assessed using the Cochrane RoB 2.0 tool (18). Domains included randomization, deviations from intended intervention, missing outcome data, outcome measurement, and selective reporting. Trials were rated as low risk if all domains were low, or as “some concerns” if any single domain received that designation.

### 2.6 Statistical analysis

We evaluated the association between an EHMD and neonatal survival of VLBW infants, compared to cow milk-containing diets. We used Forest plots to visualize the data; pooled estimates were calculated in Stata 18 (StataCorp, LLC, College Station, TX). A Sidik-Jonkman random-effect meta-regression was used to assess the relationship between effect measures (odds ratios) and the covariates birthweight and gestation (19). A random-effects model was applied to account for between-study heterogeneity, which was quantified with the *I^2^* statistic. We performed sensitivity analyses to assess these associations separately for RCTs and observational cohort studies. All studies were included in analyses, and no data were missing. A *post-hoc* sensitivity analysis was performed excluding the largest contributing study to assess its influence on the pooled estimate and its precision. Statistical significance was defined as *p* < 0.05. A pooled risk difference was not calculated because risk difference depends on the underlying baseline mortality risk, which may vary across study populations (20). Instead, an illustrative absolute difference was estimated assuming a 10% baseline mortality risk. Publication bias was visually assessed using funnel plots with the trim and fill method.

## 3 Results

### 3.1 Description of included studies and participants

**Figure 1:**
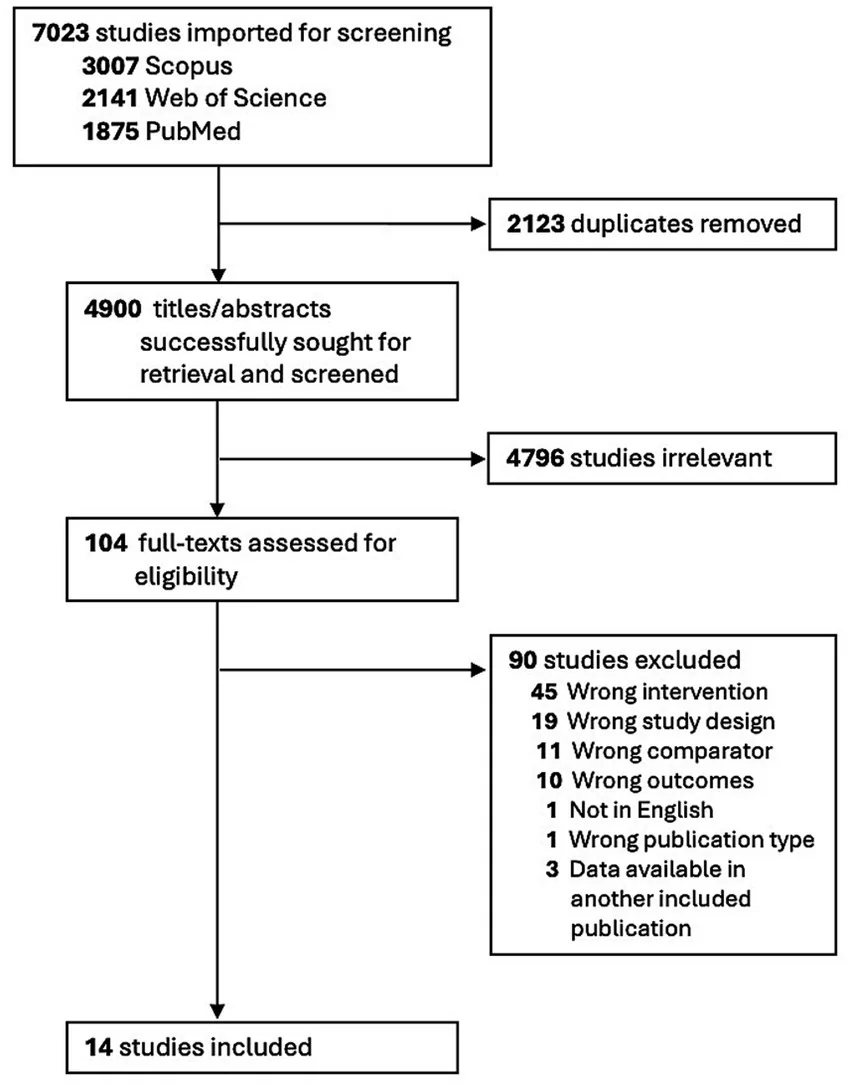
PRISMA flow diagram showing study selection.

In total 7,023 abstracts were screened, and 104 full texts were assessed for eligibility against the inclusion criteria (Figure 1). Fourteen studies met the criteria and were included in the review. The most common reasons for exclusion were inappropriate study design (e.g., reviews or case reports), use of non-vat-pasteurized human milk-derived fortifier, or absence of a cow milk-based group in the control (e.g., comparing advancement protocols using human milk-derived fortifiers in both intervention and controls groups) (7, 9, 10, 21–31).

Among the studies included, 5 were randomized trials and 9 were non-randomized cohort studies. Included studies were from high-income countries, primarily the United States but also Austria, Canada, the United Kingdom, and Sweden.

The overall study population comprised 4,713 infants born between 2004 and 2021. Of these, 50.1% were in the EHMD intervention group, while 49.9% received cow milk-derived nutritional products. The median (IQR) sample size of included studies was 204 (126, 399) with the largest study including 1,587 infants. Across included studies, enrolled cohorts were predominantly extremely preterm and very low birthweight. Mean birthweights ranged from 741 to 1,361 g and mean gestational ages ranging from 25.5 to 29.7 weeks (Table 1).

Fortification practices varied widely both within and across studies, as did the level of reporting details. In the EHMD group, fortification was initiated at enteral volumes ranging from 40 to 150 mL/kg/day, with caloric density ranging from 4 to 8 kcal/oz. and adjustments based on growth (Table 1). Earlier studies commonly began fortification at 4 kcal/oz., while more recent protocols tended to initiate at 6 kcal/oz. In contrast, control group protocols frequently started fortification at lower starting caloric density (2–4 kcal/oz), and sometimes at higher volumes than the EHMD intervention group. Use of preterm infant formula in control groups was more prevalent in earlier studies, particularly those published before 2015. Advancement strategies were more consistently reported in EHMD protocols.

### 3.2 EHMD and survival to hospital discharge

**Figure 2:**
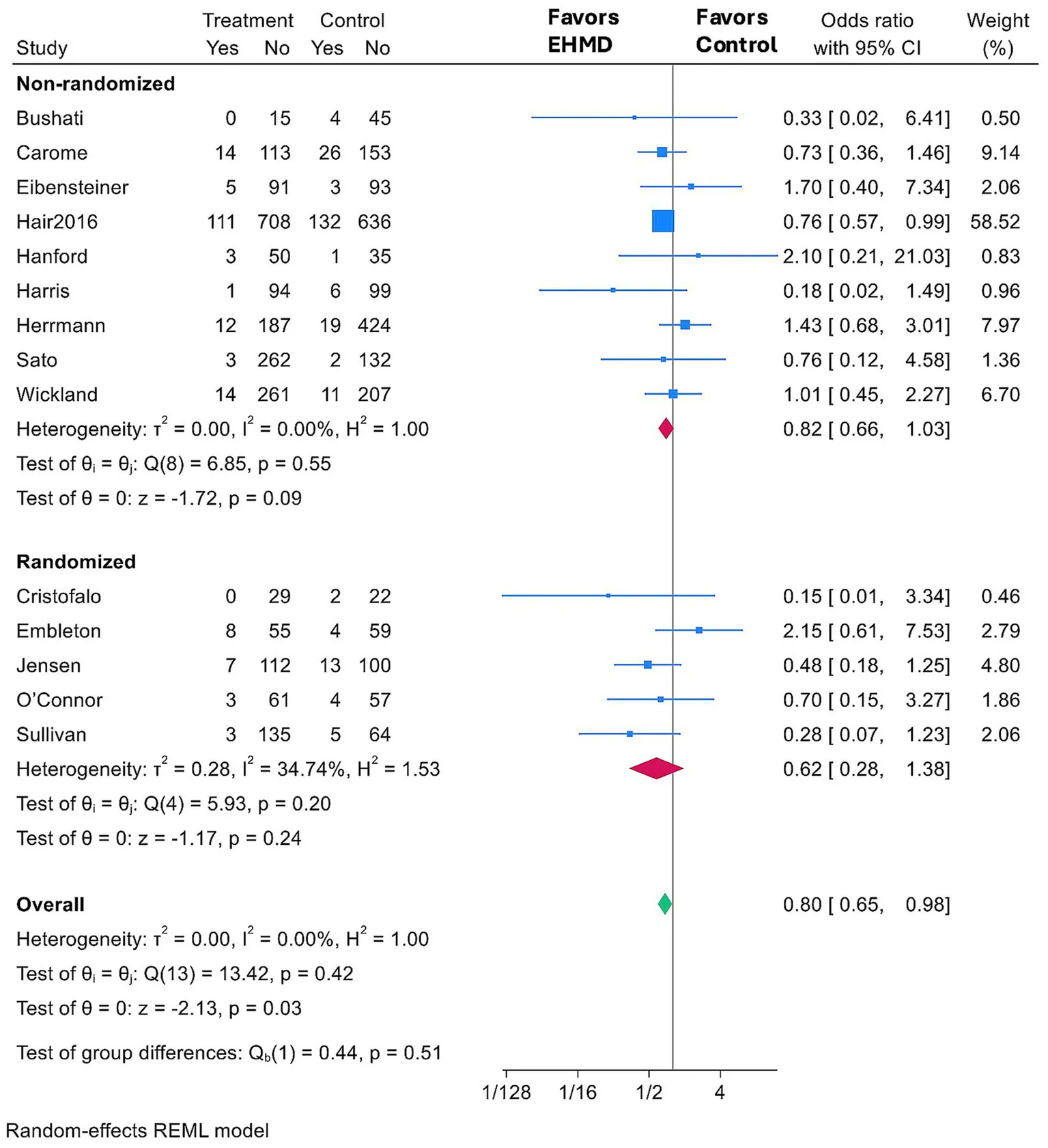
Forest plot of the association between an exclusive human milk diet (EHMD) and survival until discharge in VLBW infants. Results are presented separately by study design, with pooled estimates calculated using a random-effects model. Effect sizes are expressed as odds ratios (OR) with 95% confidence intervals. Diamonds represent pooled estimates for each subgroup and overall. The vertical line indicates the line of no effect (OR = 1).

Fourteen studies (5 randomized and 9 non-randomized) were included in the meta-analysis (Figure 2) (9, 10, 21–31). In randomized trials (7, 10, 28–31), an EHMD was associated with a non-significant reduction in the odds of death before discharge compared with cow milk-containing diets (pooled OR 0.62, 95% CI 0.28–1.38; *I^2^* = 34.7%, *p* = 0.24). In the two randomized trials directly comparing human milk-derived and cow milk-derived fortifiers (10, 30), mortality estimates were directionally similar, but no significant difference was observed between groups.

In non-randomized studies (7, 9, 21–27), the pooled effect also favored EHMD, but no significant difference emerged between groups (pooled OR 0.82, 95% CI 0.66–1.03; *I^2^* = 0%, *p* = 0.09). However, when looking at the totality of evidence, the overall pooled analysis across all study designs indicated a statistically significant association between EHMD and reduced mortality before hospital discharge (pooled OR 0.80, 95% CI 0.65–0.98; *I^2^* = 0%, tau^2^ = 0.00, *p* = 0.03). Assuming a baseline mortality of 10%, this corresponded to an estimated absolute difference of approximately 1.8 fewer deaths per 100 VLBW infants treated. There was no evidence of heterogeneity between subgroups (*p* = 0.51). In a *post-hoc* sensitivity analysis excluding Hair et al. (7), the pooled estimate was OR 0.85 (95% CI 0.60–1.20, *p* = 0.36; *I^2^* = 6.9%). The corresponding non-randomized subgroup estimate was OR 0.97 (95% CI 0.66–1.43) (Supplementary Figure 1).

Meta-regression analyses using random-effects REML models were conducted to evaluate whether mean birthweight or mean gestational age modified the association between diet and mortality before discharge. In the birthweight model (*n* = 10 studies), neither treatment nor control mean birthweight was significantly associated with effect size (Wald *χ*^2^ = 3.11, *p* = 0.21). Similarly, in the gestational age model (*n* = 9 studies), neither treatment nor control mean gestational age was significantly associated with effect size (Wald *χ*^2^ = 0.49, *p* = 0.78). Residual heterogeneity was low in both models, and inclusion of these covariates did not materially alter pooled estimates.

### 3.3 Quality assessment and publication bias

Visual inspection of the funnel plot did not suggest asymmetry (Supplementary Figure 2). Risk of bias was assessed using the RoB-NObs tool for non-randomized cohort studies and the RoB 2.0 tool for randomized controlled trials (RCTs) (Tables 2, 3). Among non-randomized cohort studies (*n* = 9), overall risk of bias ratings was *moderate* in three, *serious* in four, and *critical* in two studies. None of the non-randomized cohort studies received an overall rating of *low* bias. Common sources of bias included confounding (Domain 1), classification of exposures (Domain 3), and selection of reported results (Domain 7). Several studies demonstrated moderate-to-serious risk in multiple domains, resulting in overall downgrading to serious or critical risk.

**Table 2:** Quality assessment for non-randomized studies.

Study	Overall	Confounding	Selection of participants	Classification of exposures	Departures from intended exposures	Missing data	Measurement of outcomes	Selection of reported results
Bushati et al. (22)	SR	SR	LR	LR	MR	LR	LR	MR
Carome et al. (23)	SR	MR	LR	LR	SR	LR	LR	MR
Eibensteiner et al. (24)	MR	MR	LR	LR	LR	LR	MR	LR
Hair et al. (7)	SR	MR	MR	LR	MR	LR	MR	MR
Hanford et al. (25)	MR	MR	LR	LR	LR	LR	LR	MR
Harris et al. (21)	SR	SR	LR	MR	MR	LR	MR	NI
Herrmann and Carroll (26)	CR	SR	MR	MR	LR	MR	MR	LR
Sato et al. (9)	CR	SR	MR	LR	SR	SR	MR	MR
Wickland et al. (27)	MR	MR	LR	LR	LR	LR	MR	MR

LR, low risk (green); MR, moderate risk (tan); SR, serious risk (light orange); CR, critical risk (dark orange); NI, no information (white).

**Table 3:** Quality assessment for randomized studies.

Citation	Overall	Randomization	Deviation from intended intervention	Missing outcome data	Measurement of the outcome	Selection of the reported result
Cristofalo et al. (28)	LR	LR	LR	LR	LR	LR
Embleton et al. (29)	SC	SC	LR	LR	LR	LR
Jensen et al. (30)	LR	LR	LR	LR	LR	LR
O’Connor et al. (10)	LR	LR	LR	LR	LR	LR
Sullivan et al. (31)	LR	LR	LR	LR	LR	LR

LR, low risk (green); SC, some concerns (tan).

For RCTs (*n* = 5), four were rated as *low* risk of bias, and one as *some concerns*. Domain-specific concerns in RCTs arose in the randomization process (Domain 1). No trial was judged to be at *high* risk in all domains, and concerns were generally limited to the one area.

## 4 Discussion

In this systematic review and meta-analysis of randomized and non-randomized studies, the pooled estimate suggested an EHMD was associated with approximately 20% lower odds of death before discharge compared to cow milk–containing diets, corresponding to an estimated absolute difference of approximately 1.8 deaths per 100 infants. Subgroup analyses restricted to randomized and non-randomized studies independently did not demonstrate statistically significant differences; however, effect estimates were directionally consistent across study designs, and no heterogeneity between designs was observed. These findings should be interpreted as associative rather than causal given the inclusion of non-randomized evidence, heterogeneity in comparator feeding strategies, and the moderate to critical risk of bias identified in several observational studies. Additional adequately powered randomized or pragmatic studies would strengthen confidence in the observed association.

Several prior systematic reviews have evaluated the association between EHMD and mortality among preterm infants, though these analyses were restricted to randomized trials (6, 32–35). Mortality estimates in these reviews were typically derived from small numbers of studies and events. For example, Galis et al. (6) included four trials reporting mortality (RR 0.50, 95% CI 0.26–0.94), whereas earlier reviews included between one and four trials contributing mortality data and reported effect estimates ranging from RR 0.40 to RR 0.83, with confidence intervals that frequently included the null (32–35). The direction and magnitude of these estimates are broadly comparable to the approximately 20% lower odds of death observed in the present pooled analysis. The larger pooled sample in the present study resulted in narrower confidence intervals around the mortality estimate relative to prior reviews.

Mortality among VLBW infants is clinically important but statistically challenging to evaluate in individual feeding trials because event rates are relatively low and mortality is infrequently specified as a primary endpoint (11). Given that the median size of included studies was 204 infants, many individual trials may have been underpowered to detect modest but clinically meaningful differences in survival. Inclusion of non-randomized studies increased the number of observed events and improved estimate precision. Sensitivity analysis illustrated this point. When the largest study was excluded, removing roughly a third of the pooled cohort, the estimate changed little (OR 0.85 vs. 0.80), but the confidence interval widened to the point that no significant difference remained. Heterogeneity stayed low, and the excluded study’s own estimate was close to the pooled value, so the change is consistent with the loss of accrued events rather than distortion of the estimate by one study. Within the non-randomized studies, removal of this study attenuated the estimate towards the null (OR 0.97). The reason for this shift is unclear and may reflect variation in fortification timing, advancement, and comparator composition across the non-randomized cohorts. These considerations highlight the need for larger collaborative studies or pragmatic trial designs capable of evaluating rare but clinically important outcomes such as mortality.

Mortality is also infrequently incorporated into composite clinical outcomes because event counts in individual studies are low. For example, Abrams et al. (8), combined data from two clinical trials and reported mortality rates of 2% in the EHMD group compared with 8% in the control group, which had cow milk-containing diets (*n* = 260; *p* = 0.004). In the original trials, however, death was incorporated into composite outcomes because event counts were low (28, 31). In the present analysis, the pooled number needed to treat to prevent one death was ~55–56. Given the rarity of mortality events in this population, interpretation may benefit from consideration of the totality of available evidence, including both randomized and observational data, while recognizing the limitations inherent to each design.

Interpretation of pooled mortality estimates is further complicated by heterogeneity in feeding protocols across studies. Differences in the timing of fortification initiation, advancement of enteral feeds, and cumulative human milk exposure may impact observed associations (36–38). However, these factors may be difficult to control, even in randomized trials. For example, in an unblinded RCT, Jensen et al. (30) reported significantly later fortification in the cow milk-containing control group compared with the EHMD group (average 92 vs. 83 mL/kg/d, respectively, *p* = 0.01). Such differences may complicate interpretation of measured effect estimates by altering the cumulative enteral nutrition exposure between study arms.

Comparator diets also varied substantially across studies. Consistent with published literature describing variation in neonatal feeding practices across institutions (39), control groups differed in the degree of exposure to cow milk-derived fortifier, preterm formula, and human milk. In some studies, comparison reflected a head-to-head evaluation of human milk-derived and cow milk-derived fortifiers, whereas in others the comparator diet included varying degrees of preterm infant formula exposure. Although formula use has declined in many contemporary NICUs, it remains part of feeding protocols in some settings and therefore reflects an important component of real-world clinical practice (40). When comparator groups receive high proportions of human milk or limited exposure to cow milk-derived products, the nutritional contrast between study arms may be reduced. Accordingly, heterogeneity in comparator composition represents an important source of clinical heterogeneity when interpreting pooled mortality estimates. Further investigation may help clarify the extent to which differences in comparator diet composition influence observed associations between EHMD exposure and mortality outcomes.

Two randomized trials evaluated a direct head-to-head comparison of human milk-derived and cow milk-derived fortifiers (10, 30). These trials individually demonstrated non-significant differences in mortality, consistent with the direction of the pooled estimate but limited by small sample sizes and low event counts. Because only two such trials exist, and because mortality is a rare outcome, the available evidence is insufficient to support a robust, standalone subgroup meta-analysis. Additional adequately powered head-to-head trials are needed to more clearly evaluate fortifier-specific effects independent of broader feeding protocol differences.

All included studies evaluated vat-pasteurized human milk-derived fortifiers, reflecting our PROSPERO-registered protocol. This criterion was prespecified because these fortifiers, currently produced by a single manufacturer (Prolacta Bioscience), are the only human milk-derived fortifiers on the market that provide concentrated protein and added micronutrients required to meet the nutritional needs of VLBW infants (5, 41). Emerging human milk-derived fortifiers include powdered products that lack this concentrated protein and micronutrient enrichment (42–44) and are therefore not appropriate to meet the nutritional requirements of VLBW infants. As these represent a distinct product category, the present findings should not be extrapolated to them.

Finally, the practical and ethical challenges of conducting large, randomized trials in this population should be acknowledged. Integrating evidence from randomized and non-randomized studies may provide a pragmatic approach to improve estimation of rare clinical outcomes that are difficult to evaluate in randomized trials alone (45–47). Decisions regarding enteral feeding strategies, including the use of human milk-derived vs. cow milk-derived fortifiers, should therefore be considered within the broader context of neonatal nutrition practices, donor milk access, and institutional feeding protocols.

A strength of this review is the inclusion of both randomized and non-randomized evidence, enabling assessment of the totality of available data and increasing applicability to real-world practice. Including studies where control groups received preterm formula reflects the diversity of feeding strategies currently used in NICUs throughout the world and captures settings often excluded from prior systematic reviews. The large, combined sample size improves statistical power to evaluate a relatively rare outcome such as survival to discharge. Effect estimates were directionally concordant across RCTs and observational studies, with no evidence of between-subgroup heterogeneity, consistent with a common underlying association.

This review also has limitations. The inclusion of observational studies increased the overall sample size but introduces the potential for residual confounding related to clinician indication, center-level feeding practices, or unmeasured factors. Fortification protocols, timing of initiation, and escalation strategies varied considerably between studies and were often more clearly described for EHMD than for control groups. Such differences may influence outcomes independently of fortifier type. Inclusion of studies with formula in the control group likely introduces heterogeneity in nutrient and bioactive exposure. Most included studies were retrospective, non-randomized cohorts. With 14 included studies, the statistical power of funnel plots to detect publication bias is limited. Evidence quality varied across study designs. None of the observational studies were rated as low risk of bias, and several were rated as moderate, serious, or critical. Hair et al. (7) contributed substantial weight to the non-randomized pooled estimate and was rated as having serious risk of bias, representing an important source of uncertainty. Pooled results combining randomized and observational studies should therefore be interpreted cautiously pending higher-quality data. Finally, we synthesized heterogeneous intervention definitions and comparator exposures using a conventional random-effects framework rather than a network meta-analysis, which could more formally model indirect comparisons and varying intervention nodes among varying study designs (48). Given the limited number of studies and variability in intervention definitions in the present meta-analysis, such an approach would likely have been unstable here. We therefore used conventional meta-analysis, recognizing the limited ability to formally separate the effects of differing comparator compositions (e.g., cow milk-derived fortifier vs. formula). Although this is a methodological consideration when interpreting pooled estimates across heterogeneous feeding strategies, our findings were directionally consistent with those of Campbell-Yeo et al. (35) who conducted a network meta-analysis and reported a similar magnitude of association in a head-to-head comparison of human milk- and cow milk-based fortifiers.

This systematic review and meta-analysis suggests that an EHMD was associated with approximately 20% lower odds of death before discharge compared with cow milk–containing diets, corresponding to an estimated absolute difference of approximately 1.8 deaths per 100 infants. Effect estimates were directionally consistent across randomized and non-randomized study designs, although confidence in the observed association remains limited by imprecision in randomized studies, heterogeneity in comparator feeding strategies, and the moderate to critical risk of bias identified in several observational studies. Given the rarity of mortality as an endpoint in contemporary NICU care and the practical barriers to very large, randomized trials in this population, further evaluation will likely require pragmatic designs or large collaborative datasets. Continued efforts to standardize exposure definitions and comparator characterization may improve interpretability across future studies.

## Data Availability

The raw data supporting the conclusions of this article will be made available by the authors, upon request.
