# Supplementary material for: Exclusive human milk diet and mortality before discharge in premature and very low birthweight infants: a systematic review and meta-analysis
Source: Front Nutr. 2026 Aug 21;13:1898548. doi: 10.3389/fnut.2026.1898548 (PMC13541452; doi:10.3389/fnut.2026.1898548)
Supplement: Supplementary file 1 [file Table_1.DOCX]

Supplementary Material

# 1 Supplementary Figures and Tables

## 1.1 Supplementary Figures

**Supplementary Figure 2.** Leave-it-out analysis, excluding Hair et al (2016)[91]. Forest plot of the association between an exclusive human milk diet (EHMD) and survival until discharge in VLBW infants (n=3,128).

**Supplementary Figure 2.** Funnel plot to assess publication bias

## 1.2 Supplementary Tables

**Supplementary Table 1.** Search Strategy for PubMed, Web of Science, and Scopus

| **Database** | **Search terms** | **Results** |
| --- | --- | --- |
| **PubMed** | 1. "Milk*"[Mesh] OR "Milk, Human"[Mesh] OR "Infant Food"[Mesh] OR "Infant Formula"[Mesh] OR "Food, Fortified*"[Mesh] OR "human milk"[tw] OR "breast milk"[tw] OR “breastmilk”[tw] OR "donor human milk"[tw] OR "human milk fortifier"[tw] OR "exclusive human milk*"[tw] OR "enteral feeding"[tw] OR “enteral nutrition”[tw] | -- |
|  | 2. "Infant Death" [Mesh] OR "Death"[Mesh] OR "mortality" [tw] OR "survival to discharge" [tw] OR "survive*" [tw] OR "death"[tw] OR "necrotizing enterocolitis"[tw] OR “Enterocolitis, Necrotizing*”[Mesh] | -- |
|  | 3. "Humans"[Mesh] OR "Female"[Mesh] OR "Male"[Mesh] OR "Infant, Premature"[Mesh] OR "Infant, Extremely Low Birth Weight"[Mesh] OR "Infant, Very Low Birth Weight"[Mesh] OR "Infant, Newborn"[Mesh] OR "premature infant*"[tw] OR "preterm infant*"[tw] OR "low birthweight"[tw] | -- |
|  | #1 AND #2 AND #3 | -- |
|  | 5. "Retrospective Studies"[Mesh] OR "Epidemiologic Studies"[Mesh] OR "Clinical Trial" [Publication Type] OR "Randomized Controlled Trial" [Publication Type] OR "Published Erratum" [Publication Type] OR "retrospective stud*"[tw] OR “retrospective cohort*”[tw] OR "controlled trial"[tw] OR "randomized clinical trial"[tw] OR "observational study"[tw] OR "multi-center retrospective cohort study"[tw] OR “multicenter*” OR “single-arm*”[tw] | -- |
|  | **#1 AND #3 AND #2 AND #5** | **1,875** |
| **Web of Science** | 1. TS=("Milk" OR "Infant Food" OR "Infant Formula" OR "human milk" OR "breast milk" OR (mothers NEAR/3 milk) OR "breast feeding" OR "donor human milk" OR "donor breast milk" OR "human milk fortifier" OR "exclusive human milk" OR "enteral feeding") | -- |
|  | 2. TS=("necrotizing enterocolitis" OR "medical NEC" OR "surgical NEC" OR "feeding intolerance" OR "feeding interruption") | -- |
|  | 3. TS=(Humans OR Female OR Male OR "Extremely Low Birth Weight" OR "infant, extremely low birthweight" OR "Very Low Birth Weight" OR "infant, very low birthweight" OR "Newborn Infant" OR "premature infant" OR "preterm infant" OR "low birthweight") | -- |
|  | **#1 AND #2 AND #3** | **2,141** |
| **Scopus** | (TITLE-ABS-KEY(Milk OR "Milk, Human" OR "Infant Food" OR "Infant Formula" OR "Food, Fortified" OR "human milk" OR "breast milk" OR breastmilk OR "donor human milk" OR "human milk fortifier" OR "exclusive human milk" OR "enteral feeding")) | -- |
|  | (TITLE-ABS-KEY("Infant, Premature, Diseases" OR "Enterocolitis, Necrotizing" OR "necrotizing enterocolitis" OR "medical NEC" OR "surgical NEC" OR "feeding intolerance" OR "feeding interruption" OR death OR survival)) | -- |
|  | (TITLE-ABS-KEY(Humans OR Female OR Male OR "Infant, Premature" OR "Infant, Extremely Low Birth Weight" OR "Infant, Very Low Birth Weight" OR "Infant, Newborn" OR "premature infant" OR "preterm infant" OR "low birthweight")) | -- |
|  | (TITLE-ABS-KEY("Retrospective Studies" OR "Epidemiologic Studies" OR "Clinical Trial" OR "Randomized Controlled Trial" OR "Published Erratum" OR "retrospective study" OR "controlled trial" OR "randomized clinical trial" OR "observational study" OR "multi-center retrospective cohort study")) | -- |
|  | **#1 AND #2 AND #3 AND #4** | **3,007** |

**Supplementary Table 2. Excluded studies (n=90)**

| **References** | **Reason for Exclusion** |
| --- | --- |
| [1-45] | Wrong intervention (n=45) |
| [46-64] | Wrong study design (n=19) |
| [65-75] | Wrong comparator (n=11) |
| [76-85] | Wrong outcomes (n=10) |
| [86] | Not in English (n=1) |
| [87] | Wrong publication type (n=1) |
| [88-90] | Data available in another included publication (n=3) |

**REFERENCES**

1.	Zachariassen, G.; Fenger-Gron, J. Preterm dietary study: meal frequency, regurgitation and the surprisingly high use of laxatives among formula-fed infants following discharge. *Acta Paediatr* **2014**, *103*, e116-122, doi:10.1111/apa.12524.

2.	Adhisivam, B.; Kohat, D.; Tanigasalam, V.; Bhat, V.; Plakkal, N.; Palanivel, C. Does fortification of pasteurized donor human milk increase the incidence of necrotizing enterocolitis among preterm neonates? A randomized controlled trial. *J Matern Fetal Neonatal Med* **2019**, *32*, 3232-3237, doi:10.1080/14767058.2018.1461828.

3.	Alyahya, W.; Simpson, J.; Garcia, A.L.; Mactier, H.; Young, D.; Edwards, C.A. Association between Early Feeding Patterns and Neonatal Outcomes in Very Preterm Infants: A Retrospective Cohort Study. *Neonatology* **2023**, *120*, 71-80, doi:10.1159/000527522.

4.	Barr, P.A.; Mally, P.V.; Caprio, M.C. Standardized Nutrition Protocol for Very Low-Birth-Weight Infants Resulted in Less Use of Parenteral Nutrition and Associated Complications, Better Growth, and Lower Rates of Necrotizing Enterocolitis. *JPEN J Parenter Enteral Nutr* **2019**, *43*, 540-549, doi:10.1002/jpen.1453.

5.	Abramson, J.; Szatkowski, L.; Bains, M.; Orton, E.; Budge, H.; Spruce, M.; Ojha, S. Effects of implementation of a care bundle on rates of necrotising enterocolitis and own mother's milk feeding in the East Midlands: protocol for a mixed methods impact and process evaluation study. *BMJ Open* **2024**, *14*, e078633, doi:10.1136/bmjopen-2023-078633.

6.	Belling-Dierks, F.; Glaser, K.; Wirbelauer, J.; Rücker, V.; Frieauff, E. Does rapid enteral feeding increase intestinal morbidity in very low birth weight infants? A retrospective analysis. *J Matern Fetal Neonatal Med* **2017**, *30*, 2690-2696, doi:10.1080/14767058.2016.1261284.

7.	Berkhout, D.J.C.; Klaassen, P.; Niemarkt, H.J.; de Boode, W.P.; Cossey, V.; van Goudoever, J.B.; Hulzebos, C.V.; Andriessen, P.; van Kaam, A.H.; Kramer, B.W., et al. Risk Factors for Necrotizing Enterocolitis: A Prospective Multicenter Case-Control Study. *Neonatology* **2018**, *114*, 277-284, doi:10.1159/000489677.

8.	Bharadwaj, N.; Panigrahy, N.; Bagga, N.; Chirla, D.K. Human Milk-Derived Fortifier as Rescue Therapy in Very Preterm Infants Intolerant to Cow's Milk-Derived Fortifier. *Indian J Pediatr* **2022**, *89*, 1131-1133, doi:10.1007/s12098-022-04309-7.

9.	Butler, T.J.; Szekely, L.J.; Grow, J.L. A standardized nutrition approach for very low birth weight neonates improves outcomes, reduces cost and is not associated with increased rates of necrotizing enterocolitis, sepsis or mortality. *J Perinatol* **2013**, *33*, 851-857, doi:10.1038/jp.2013.66.

10.	Cakmak Celik, F.; Aygun, C.; Cetinoglu, E. Does early enteral feeding of very low birth weight infants increase the risk of necrotizing enterocolitis? *Eur J Clin Nutr* **2009**, *63*, 580-584, doi:10.1038/sj.ejcn.1602957.

11.	Cañizo Vázquez, D.; Salas García, S.; Izquierdo Renau, M.; Iglesias-Platas, I. Availability of Donor Milk for Very Preterm Infants Decreased the Risk of Necrotizing Enterocolitis without Adversely Impacting Growth or Rates of Breastfeeding. *Nutrients* **2019**, *11*, doi:10.3390/nu11081895.

12.	Chinnappan, A.; Sharma, A.; Agarwal, R.; Thukral, A.; Deorari, A.; Sankar, M.J. Fortification of Breast Milk With Preterm Formula Powder vs Human Milk Fortifier in Preterm Neonates: A Randomized Noninferiority Trial. *JAMA Pediatr* **2021**, *175*, 790-796, doi:10.1001/jamapediatrics.2021.0678.

13.	Christensen, R.D.; Lambert, D.K.; Gordon, P.V.; Baer, V.L.; Gerday, E.; Henry, E. Neonates presenting with bloody stools and eosinophilia can progress to two different types of necrotizing enterocolitis. *J Perinatol* **2012**, *32*, 874-879, doi:10.1038/jp.2011.163.

14.	Colaizy, T.T.; Poindexter, B.B.; McDonald, S.A.; Bell, E.F.; Carlo, W.A.; Carlson, S.J.; DeMauro, S.B.; Kennedy, K.A.; Nelin, L.D.; Sánchez, P.J., et al. Neurodevelopmental Outcomes of Extremely Preterm Infants Fed Donor Milk or Preterm Infant Formula: A Randomized Clinical Trial. *Jama* **2024**, *331*, 582-591, doi:10.1001/jama.2023.27693.

15.	Corpeleijn, W.E.; de Waard, M.; Christmann, V.; van Goudoever, J.B.; Jansen-van der Weide, M.C.; Kooi, E.M.; Koper, J.F.; Kouwenhoven, S.M.; Lafeber, H.N.; Mank, E., et al. Effect of Donor Milk on Severe Infections and Mortality in Very Low-Birth-Weight Infants: The Early Nutrition Study Randomized Clinical Trial. *JAMA Pediatr* **2016**, *170*, 654-661, doi:10.1001/jamapediatrics.2016.0183.

16.	Corpeleijn, W.E.; Kouwenhoven, S.M.; Paap, M.C.; van Vliet, I.; Scheerder, I.; Muizer, Y.; Helder, O.K.; van Goudoever, J.B.; Vermeulen, M.J. Intake of own mother's milk during the first days of life is associated with decreased morbidity and mortality in very low birth weight infants during the first 60 days of life. *Neonatology* **2012**, *102*, 276-281, doi:10.1159/000341335.

17.	Cortez, J.; Makker, K.; Kraemer, D.F.; Neu, J.; Sharma, R.; Hudak, M.L. Maternal milk feedings reduce sepsis, necrotizing enterocolitis and improve outcomes of premature infants. *J Perinatol* **2018**, *38*, 71-74, doi:10.1038/jp.2017.149.

18.	D'Costa, R.; Fucile S PhD, O.T.; Dickson, B.R.; Gallipoli, A.; Dow Ke Md, F. Benefits of a Standardized Enteral Feeding Protocol on the Nutrition and Health Outcomes of Very Low Birth Weight Preterm Infants. *Can J Diet Pract Res* **2022**, *83*, 147-150, doi:10.3148/cjdpr-2022-003.

19.	Dritsakou, K.; Liosis, G.; Valsami, G.; Polychronopoulos, E.; Skouroliakou, M. Improved outcomes of feeding low birth weight infants with predominantly raw human milk versus donor banked milk and formula. *J Matern Fetal Neonatal Med* **2016**, *29*, 1131-1138, doi:10.3109/14767058.2015.1038232.

20.	Fortmann, I.; Marißen, J.; Siller, B.; Spiegler, J.; Humberg, A.; Hanke, K.; Faust, K.; Pagel, J.; Eyvazzadeh, L.; Brenner, K., et al. Lactobacillus Acidophilus/Bifidobacterium Infantis Probiotics Are Beneficial to Extremely Low Gestational Age Infants Fed Human Milk. *Nutrients* **2020**, *12*, doi:10.3390/nu12030850.

21.	Gates, A.; Thompson, A.B.; Marin, T.; Waller, J.L.; Patel, J.; Stansfield, B.K. Novel multinutrient human milk-based human milk fortifier promotes growth and tolerance in premature infants. *JPEN J Parenter Enteral Nutr* **2022**, *46*, 817-827, doi:10.1002/jpen.2249.

22.	Gephart, S.M.; Quinn, M.C. Relationship of Necrotizing Enterocolitis Rates to Adoption of Prevention Practices in US Neonatal Intensive Care Units. *Adv Neonatal Care* **2019**, *19*, 321-332, doi:10.1097/anc.0000000000000592.

23.	Gregory, K.E. Clinical predictors of necrotizing enterocolitis in premature infants. *Nurs Res* **2008**, *57*, 260-270, doi:10.1097/01.NNR.0000313488.72035.a9.

24.	Hällström, M.; Koivisto, A.M.; Janas, M.; Tammela, O. Frequency of and risk factors for necrotizing enterocolitis in infants born before 33 weeks of gestation. *Acta Paediatr* **2003**, *92*, 111-113, doi:10.1111/j.1651-2227.2003.tb00479.x.

25.	Hammond, P.J.; Flett, M.E.; De La Hunt, M. Fulminant necrotising enterocolitis immediately following change to low birth weight formula feeds. *Eur J Pediatr Surg* **2008**, *18*, 185-187, doi:10.1055/s-2008-1038440.

26.	Hanson, C.; Sundermeier, J.; Dugick, L.; Lyden, E.; Anderson-Berry, A.L. Implementation, process, and outcomes of nutrition best practices for infants <1500 g. *Nutr Clin Pract* **2011**, *26*, 614-624, doi:10.1177/0884533611418984.

27.	Hemmati, F.; Ghassemzadeh, M. The Effect of Oral Protein Supplementation on the Growth of Very Low Birth Weight Preterm Infants Admitted to the Neonatal Intensive Care Unit: A Randomized Clinical Trial. *J Mother Child* **2023**, *27*, 21-29, doi:10.34763/jmotherandchild.20232701.d-22-00072.

28.	Hogewind-Schoonenboom, J.E.; Rövekamp-Abels, L.W.W.; de Wijs-Meijler, D.P.M.; Maduro, M.D.; Jansen-van der Weide, M.C.; van Goudoever, J.B.; Hulst, J.M. The Effect of Maternal Milk on Tolerance and Growth in Premature Infants: A Hypothesis-generating Study. *J Pediatr Gastroenterol Nutr* **2017**, *64*, 971-974, doi:10.1097/mpg.0000000000001427.

29.	Kanmaz, H.G.; Mutlu, B.; Canpolat, F.E.; Erdeve, O.; Oguz, S.S.; Uras, N.; Dilmen, U. Human milk fortification with differing amounts of fortifier and its association with growth and metabolic responses in preterm infants. *J Hum Lact* **2013**, *29*, 400-405, doi:10.1177/0890334412459903.

30.	Klingenberg, C.; Muraas, F.K.; Isaksen, C.E.; Nilsen, T.; Torgersen, M.; Melum-Hansen, C. Growth and neurodevelopment in very preterm infants receiving a high enteral volume-feeding regimen - a population-based cohort study. *J Matern Fetal Neonatal Med* **2019**, *32*, 1664-1672, doi:10.1080/14767058.2017.1414796.

31.	Kombo, L.; Smith, J.; Van Wyk, L. Somatic Growth of Enteral-Only Fed Extremely Low Birth Weight Infants in a Resource-Restricted Setting. *J Trop Pediatr* **2021**, *67*, doi:10.1093/tropej/fmaa119.

32.	Le, V.T.; Klebanoff, M.A.; Talavera, M.M.; Slaughter, J.L. Transient effects of transfusion and feeding advances (volumetric and caloric) on necrotizing enterocolitis development: A case-crossover study. *PLoS One* **2017**, *12*, e0179724, doi:10.1371/journal.pone.0179724.

33.	Ma, X.W.; Fan, W.Q. Earlier Nutrient Fortification of Breastmilk Fed LBW Infants Improves Jaundice Related Outcomes. *Nutrients* **2020**, *12*, doi:10.3390/nu12072116.

34.	Masoli, D.; Mena, P.; Dominguez, A.; Ramolfo, P.; Vernal, P.; Pantoja, M.A.; Esparza, R.; Hübner, M.E.; Ríos, A.; Faunes, M., et al. Growth of Very Low Birth Weight Infants Who Received a Liquid Human Milk Fortifier: A Randomized, Controlled Multicenter Trial. *J Pediatr Gastroenterol Nutr* **2022**, *74*, 424-430, doi:10.1097/mpg.0000000000003321.

35.	Moya, F.; Sisk, P.M.; Walsh, K.R.; Berseth, C.L. A new liquid human milk fortifier and linear growth in preterm infants. *Pediatrics* **2012**, *130*, e928-935, doi:10.1542/peds.2011-3120.

36.	Murthy, S.; Parker, P.R.; Gross, S.J. Low rate of necrotizing enterocolitis in extremely low birth weight infants using a hospital-based preterm milk bank. *J Perinatol* **2019**, *39*, 108-114, doi:10.1038/s41372-018-0235-3.

37.	Parm, Ü.; Metsvaht, T.; Ilmoja, M.L.; Lutsar, I. Gut colonization by aerobic microorganisms is associated with route and type of nutrition in premature neonates. *Nutr Res* **2015**, *35*, 496-503, doi:10.1016/j.nutres.2015.04.006.

38.	Patel, S.; Chaudhari, M.; Kadam, S.; Rao, S.; Patole, S. Standardized feeding and probiotic supplementation for reducing necrotizing enterocolitis in preterm infants in a resource limited set up. *Eur J Clin Nutr* **2018**, *72*, 281-287, doi:10.1038/s41430-017-0040-7.

39.	Peng, W.; Han, J.; Li, S.; Zhang, L.; Yang, C.; Guo, J.; Cao, Y. The Association of Human Milk Feeding With Short-Term Health Outcomes Among Chinese Very/Extremely Low Birth Weight Infants. *J Hum Lact* **2022**, *38*, 670-677, doi:10.1177/08903344221078237.

40.	Reali, A.; Greco, F.; Marongiu, G.; Deidda, F.; Atzeni, S.; Campus, R.; Dessì, A.; Fanos, V. Individualized fortification of breast milk in 41 Extremely Low Birth Weight (ELBW) preterm infants. *Clin Chim Acta* **2015**, *451*, 107-110, doi:10.1016/j.cca.2015.04.027.

41.	Rochow, N.; Fusch, G.; Ali, A.; Bhatia, A.; So, H.Y.; Iskander, R.; Chessell, L.; El Helou, S.; Fusch, C. Individualized target fortification of breast milk with protein, carbohydrates, and fat for preterm infants: A double-blind randomized controlled trial. *Clin Nutr* **2021**, *40*, 54-63, doi:10.1016/j.clnu.2020.04.031.

42.	Sisk, P.M.; Lambeth, T.M.; Rojas, M.A.; Lightbourne, T.; Barahona, M.; Anthony, E.; Auringer, S.T. Necrotizing Enterocolitis and Growth in Preterm Infants Fed Predominantly Maternal Milk, Pasteurized Donor Milk, or Preterm Formula: A Retrospective Study. *Am J Perinatol* **2017**, *34*, 676-683, doi:10.1055/s-0036-1597326.

43.	Stefanescu, B.M.; Gillam-Krakauer, M.; Stefanescu, A.R.; Markham, M.; Kosinski, J.L. Very low birth weight infant care: adherence to a new nutrition protocol improves growth outcomes and reduces infectious risk. *Early Hum Dev* **2016**, *94*, 25-30, doi:10.1016/j.earlhumdev.2016.01.011.

44.	Trang, S.; Zupancic, J.A.F.; Unger, S.; Kiss, A.; Bando, N.; Wong, S.; Gibbins, S.; O'Connor, D.L. Cost-Effectiveness of Supplemental Donor Milk Versus Formula for Very Low Birth Weight Infants. *Pediatrics* **2018**, *141*, doi:10.1542/peds.2017-0737.

45.	Yigit, S.; Akgoz, A.; Memisoglu, A.; Akata, D.; Ziegler, E.E. Breast milk fortification: effect on gastric emptying. *J Matern Fetal Neonatal Med* **2008**, *21*, 843-846, doi:10.1080/14767050802287176.

46.	Fleig, L.; Hagan, J.; Lee, M.L.; Abrams, S.A.; Hawthorne, K.M.; Hair, A.B. Growth outcomes of small for gestational age preterm infants before and after implementation of an exclusive human milk-based diet. *J Perinatol* **2021**, *41*, 1859-1864, doi:10.1038/s41372-021-01082-x.

47.	Ganapathy, V.; Hay, J.W.; Kim, J.H. Costs of necrotizing enterocolitis and cost-effectiveness of exclusively human milk-based products in feeding extremely premature infants. *Breastfeed Med* **2012**, *7*, 29-37, doi:10.1089/bfm.2011.0002.

48.	Hair, A.B.; Hawthorne, K.M.; Chetta, K.E.; Abrams, S.A. Human milk feeding supports adequate growth in infants ≤ 1250 grams birth weight. *BMC Res Notes* **2013**, *6*, doi:10.1186/1756-0500-6-459.

49.	Hair, A.B.; Rechtman, D.J.; Lee, M.L.; Niklas, V. Beyond Necrotizing Enterocolitis: Other Clinical Advantages of an Exclusive Human Milk Diet. *Breastfeed Med* **2018**, *13*, 408-411, doi:10.1089/bfm.2017.0192.

50.	Underwood, M.A.; Kalanetra, K.M.; Bokulich, N.A.; Mirmiran, M.; Barile, D.; Tancredi, D.J.; German, J.B.; Lebrilla, C.B.; Mills, D.A. Prebiotic oligosaccharides in premature infants. *J Pediatr Gastroenterol Nutr* **2014**, *58*, 352-360, doi:10.1097/mpg.0000000000000211.

51.	Vardar, G.; Ozdil, M.; Tufekci, S. Awareness or neglecting the diagnosis of cow milk protein allergy in the neonatal period. *Asia Pac J Clin Nutr* **2023**, *32*, 257-264, doi:10.6133/apjcn.202306_32(2).0008.

52.	Osmanova, M.; Müller, M.J.; Habisch, B.; Hippe, A.; Seeliger, S. Nutrition of Infants with Very Low Birth Weight using Human and Bovine Based Milk Fortifier: Benefits and Costs. *Neonatal and Pediatric Medicine* **2021**, *7*.

53.	Grace, E.; Hilditch, C.; Gomersall, J.; Collins, C.T.; Rumbold, A.; Keir, A.K. Safety and efficacy of human milk-based fortifier in enterally fed preterm and/or low birthweight infants: a systematic review and meta-analysis. *Arch Dis Child Fetal Neonatal Ed* **2021**, *106*, 137-142, doi:10.1136/archdischild-2020-319406.

54.	van Katwyk, S.; Ferretti, E.; Kumar, S.; Hutton, B.; Harrold, J.; Walker, M.; Forster, A.J.; Thavorn, K. Economic Analysis of Exclusive Human Milk Diets for High-Risk Neonates, a Canadian Hospital Perspective. *Breastfeed Med* **2020**, *15*, 377-386, doi:10.1089/bfm.2019.0273.

55.	Hampson, G.; Roberts, S.L.E.; Lucas, A.; Parkin, D. An economic analysis of human milk supplementation for very low birth weight babies in the USA. *BMC Pediatr* **2019**, *19*, 337, doi:10.1186/s12887-019-1691-4.

56.	Aoki, M.; Motoichirou, S.; Mizuno, K.; Nakano, Y.; Asai, H.; Hawthorne, K.M. First Use in Japan of Exclusive Human Milk Diet: Case Report Series. . *Ann Clin Case Rep* **2020**, *5*, 1877, doi:10.25107/2474-1655.1877.

57.	Lucas, A.; Boscardin, J.; Abrams, S.A. Preterm Infants Fed Cow's Milk-Derived Fortifier Had Adverse Outcomes Despite a Base Diet of Only Mother's Own Milk. *Breastfeed Med* **2020**, *15*, 297-303, doi:10.1089/bfm.2019.0133.

58.	Lofiego, P.; Samedi, V.; Rai, S.E. Severe feeding intolerance in extremely preterm neonates successfully treated with human milk derived human milk fortifier: A case series. *Journal of Clinical Neonatology* **2021**, *10*, doi:10.4103/jcn.jcn_210_20.

59.	Reyes, S.M.; Moore, J.B.; Lee, M.L.; Ferry, J.; Elliott, M.J. Associations of an Exclusive Human Milk Diet with Morbidity and Mortality in ELBW Infants Born Weighing <750 Grams: An Individual Participant Data Meta-analysis. *Neonatology Today* **2023**, *18*, 3-23.

60.	Scholz, S.M.; Greiner, W. An exclusive human milk diet for very low birth weight newborns-A cost-effectiveness and EVPI study for Germany. *PLoS One* **2019**, *14*, e0226496, doi:10.1371/journal.pone.0226496.

61.	Lucas, A.; Assad, M.; Sherman, J.; Boscardin, J.; Abrams, S. Safety of Cow's Milk-Derived Fortifiers Used with an All-Human Milk Base Diet in Very Low Birthweight Preterm Infants. *Neonatology Today* **2020**, *15*, 3-16.

62.	Vasu, V.; Mulla, S.; Pandya, A.; Card, D.; Shearer, M.J.; Clarke, P. Late-onset vitamin K deficiency bleeding in an extremely preterm infant fed an exclusively human milk-based diet. *J Thromb Haemost* **2023**, 10.1016/j.jtha.2023.10.029, doi:10.1016/j.jtha.2023.10.029.

63.	Philip, R.K.; Romeih, E.; Bailie, E.; Daly, M.; McGourty, K.D.; Grabrucker, A.M.; Dunne, C.P.; Walker, G. Exclusive Human Milk Diet for Extremely Premature Infants: A Novel Fortification Strategy That Enhances the Bioactive Properties of Fresh, Frozen, and Pasteurized Milk Specimens. *Breastfeed Med* **2023**, *18*, 279-290, doi:10.1089/bfm.2022.0254.

64.	Abrams, S.A.; Schanler, R.J.; Lee, M.L.; Rechtman, D.J. Greater mortality and morbidity in extremely preterm infants fed a diet containing cow milk protein products. *Breastfeed Med* **2014**, *9*, 281-285, doi:10.1089/bfm.2014.0024.

65.	Ford, S.L.; Lohmann, P.; Preidis, G.A.; Gordon, P.S.; O'Donnell, A.; Hagan, J.; Venkatachalam, A.; Balderas, M.; Luna, R.A.; Hair, A.B. Improved feeding tolerance and growth are linked to increased gut microbial community diversity in very-low-birth-weight infants fed mother's own milk compared with donor breast milk. *Am J Clin Nutr* **2019**, *109*, 1088-1097, doi:10.1093/ajcn/nqz006.

66.	Huston, R.; Lee, M.; Rider, E.; Stawarz, M.; Hedstrom, D.; Pence, M.; Chan, V.; Chambers, J.; Rogers, S.; Sager, N., et al. Early fortification of enteral feedings for infants <1250 grams birth weight receiving a human milk diet including human milk based fortifier. *J Neonatal Perinatal Med* **2020**, *13*, 215-221, doi:10.3233/NPM-190300.

67.	Hair, A.B.; Blanco, C.L.; Moreira, A.G.; Hawthorne, K.M.; Lee, M.L.; Rechtman, D.J.; Abrams, S.A. Randomized trial of human milk cream as a supplement to standard fortification of an exclusive human milk-based diet in infants 750-1250 g birth weight. *J Pediatr* **2014**, *165*, 915-920, doi:10.1016/j.jpeds.2014.07.005.

68.	Visuthranukul, C.; Abrams, S.A.; Hawthorne, K.M.; Hagan, J.L.; Hair, A.B. Premature small for gestational age infants fed an exclusive human milk-based diet achieve catch-up growth without metabolic consequences at 2 years of age. . *Arch Dis Child Fetal Neonatal Ed* **2019**, *104*, F242-F247, doi:10.1136/archdischild-2017-314547.

69.	Bergner, E.M.; Shypailo, R.; Visuthranukul, C.; Hagan, J.; O'Donnell, A.R.; Hawthorne, K.M.; Abrams, S.A.; Hair, A.B. Growth, Body Composition, and Neurodevelopmental Outcomes at 2 Years Among Preterm Infants Fed an Exclusive Human Milk Diet in the Neonatal Intensive Care Unit: A Pilot Study. *Breastfeed Med* **2020**, *15*, 304-311, doi:10.1089/bfm.2019.0210.

70.	Rahman, A.; Kase, J.S.; Murray, Y.L.; Parvez, B. Neurodevelopmental Outcome of Extremely Low Birth Weight Infants Fed an Exclusive Human Milk Diet Is Not Affected by Growth Velocity. *Breastfeed Med* **2020**, *15*, 362-369, doi:10.1089/bfm.2019.0214.

71.	Chetta, K.E.; Hair, A.B.; Hawthorne, K.M.; Abrams, S.A. Serum phosphorus levels in premature infants receiving a donor human milk derived fortifier. *Nutrients* **2015**, *7*, 2562-2573, doi:10.3390/nu7042562.

72.	Salas, A.A.; Gunawan, E.; Nguyen, K.; Reeves, A.; Argent, V.; Finck, A.; Carlo, W.A. Early Human Milk Fortification in Infants Born Extremely Preterm: A Randomized Trial. *Pediatrics* **2023**, *152*, doi:10.1542/peds.2023-061603.

73.	Lambert, D.K.; Christensen, R.D.; Baer, V.L.; Henry, E.; Gordon, P.V.; Besner, G.E.; Wilkes, J.; Wiedmeier, S.E.; Gerday, E. Fulminant necrotizing enterocolitis in a multihospital healthcare system. *J Perinatol* **2012**, *32*, 194-198, doi:10.1038/jp.2011.61.

74.	Colacci, M.; Murthy, K.; DeRegnier, R.O.; Khan, J.Y.; Robinson, D.T. Growth and Development in Extremely Low Birth Weight Infants After the Introduction of Exclusive Human Milk Feedings. *Am J Perinatol* **2017**, *34*, 130-137, doi:10.1055/s-0036-1584520.

75.	El-Fadeel, H.; Velumula, P.; Lulic-Botica, M.; Natarajan, G.; Thomas, R.; Botica, G.; Bajaj, M. Effect of an exclusive human milk diet on feeding tolerance in preterm infants. *J Perinatol* **2022**, *42*, 1070-1075, doi:10.1038/s41372-022-01348-y.

76.	Hair, A.B.; Patel, A.L.; Kiechl-Kohlendorfer, U.; Kim, J.H.; Schanler, R.J.; Hawthorne, K.M.; Itriago, E.; Abrams, S.A.; Blanco, C.L. Neurodevelopmental outcomes of extremely preterm infants fed an exclusive human milk-based diet versus a mixed human milk + bovine milk-based diet: a multi-center study. *J Perinatol* **2022**, *42*, 1485-1488, doi:10.1038/s41372-022-01513-3.

77.	Hopperton, K.E.; O'Connor, D.L.; Bando, N.; Conway, A.M.; Ng, D.V.Y.; Kiss, A.; Jackson, J.; Ly, L.; OptiMoM Feeding Group, U., S. L. Nutrient Enrichment of Human Milk with Human and Bovine Milk-Based Fortifiers for Infants Born <1250 g: 18-Month Neurodevelopment Follow-Up of a Randomized Clinical Trial. *Curr Dev Nutr* **2019**, *3*, doi:10.1093/cdn/nzz129.

78.	Ackley, D.; Wang, H.; D'Angio, C.T.; Meyers, J.; Young, B.E. Human milk-derived fortifiers are linked with feed extension due to Hypoglycemia in infants <1250 g or <30 weeks: a matched retrospective chart review. *J Perinatol* **2023**, *43*, 624-628, doi:10.1038/s41372-023-01654-z.

79.	Holzapfel, L.F.; Hair, A.B.; Preidis, G.A.; Halder, T.; Yang, H.; Unger, J.P.; Freedman, S.; Martin, C.R. Fecal Elastase in Preterm Infants to Predict Growth Outcomes. *J Pediatr Gastroenterol Nutr* **2023**, *76*, 206-212, doi:10.1097/MPG.0000000000003672.

80.	Assad, M.; Elliott, M.J.; Abraham, J.H. Decreased cost and improved feeding tolerance in VLBW infants fed an exclusive human milk diet. *J Perinatol* **2016**, *36*, 216-220, doi:10.1038/jp.2015.168.

81.	Huston, R.K.; Markell, A.M.; McCulley, E.A.; Gardiner, S.K.; Sweeney, S.L. Improving Growth for Infants </=1250 Grams Receiving an Exclusive Human Milk Diet. *Nutr Clin Pract* **2018**, *33*, 671-678, doi:10.1002/ncp.10054.

82.	Huston, R.K.; Markell, A.M.; McCulley, E.A.; Pathak, M.; Rogers, S.P.; Sweeney, S.L.; Dolphin, N.G.; Gardiner, S.K. Decreasing Necrotizing Enterocolitis and Gastrointestinal Bleeding in the Neonatal Intensive Care Unit. *ICAN: Infant, Child, & Adolescent Nutrition* **2014**, *6*, 86-93, doi:10.1177/1941406413519267.

83.	Swanson, J.R.; Becker, A.; Fox, J.; Horgan, M.; Moores, R.; Pardalos, J.; Pinheiro, J.; Stewart, D.; Robinson, T. Implementing an exclusive human milk diet for preterm infants: real-world experience in diverse NICUs. *BMC Pediatr* **2023**, *23*, 237, doi:10.1186/s12887-023-04047-5.

84.	Tetarbe, M.; Chang, M.R.; Barton, L.; Cayabyab, R.; Ramanathan, R. Economic and Clinical Impact of Using Human Milk-Derived Fortifier in Very Low Birth Weight Infants. *Breastfeed Med* **2024**, *19*, 114-119, doi:10.1089/bfm.2023.0163.

85.	Wickland, J.; Wade, C.; Micetic, B.; Meredith, K.; Martin, G. A Retrospective Analysis of the Effects of an Exclusively Human Milk Protein Diet on Neonatal Feeding Tolerance. *Am J Perinatol* **2022**, *39*, 995-1000, doi:10.1055/s-0040-1721374.

86.	Wang, L.; Zhao, X.P.; Liu, H.J.; Deng, L.; Liang, H.; Duan, S.Q.; Yang, Y.H.; Zhang, H.Y. [Evidence-based standardized nutrition protocol can shorten the time to full enteral feeding in very preterm/very low birth weight infants]. *Zhongguo Dang Dai Er Ke Za Zhi* **2022**, *24*, 648-653, doi:10.7499/j.issn.1008-8830.2202121.

87.	Jensen, G.B.; Ahlsson, F.; Domellöf, M.; Elfvin, A.; Naver, L.; Abrahamsson, T. Nordic study on human milk fortification in extremely preterm infants: a randomised controlled trial-the N-forte trial. *BMJ Open* **2021**, *11*, e053400, doi:10.1136/bmjopen-2021-053400.

88.	Delaney Manthe, E.; Perks, P.H.; Swanson, J.R. Team-Based Implementation of an Exclusive Human Milk Diet. *Adv Neonatal Care* **2019**, *19*, 460-467, doi:10.1097/ANC.0000000000000676.

89.	Chang, M.R.; Tetarbe, M.; Barton, L.; Ramanathan, R.; Cayabyab, R. Transient Hypoglycemia and Biochemical Differences in Infants Less Than 1,250 G at Birth Fed Human Milk with Human Milk-Derived Fortifier versus Cow Milk-Derived Fortifier. *Am J Perinatol* **2023**, 10.1055/a-2164-7957, doi:10.1055/a-2164-7957.

90.	Uthaya, S.; Jeffries, S.; Andrzejewska, I.; Vasu, V.; Embleton, N.D.; Modi, N. Randomised controlled trial of human derived breast milk fortifier versus bovine milk fortifier on body composition in very preterm babies. *Early Hum Dev* **2022**, *171*, 105619, doi:10.1016/j.earlhumdev.2022.105619.

91.	Hair, A.B.; Peluso, A.M.; Hawthorne, K.M.; Perez, J.; Smith, D.P.; Khan, J.Y.; O'Donnell, A.; Powers, R.J.; Lee, M.L.; Abrams, S.A. Beyond Necrotizing Enterocolitis Prevention: Improving Outcomes with an Exclusive Human Milk-Based Diet. *Breastfeed Med* **2016**, *11*, 70-74, doi:10.1089/bfm.2015.0134.
